# Endothelial Trauma Depends on Surface Charge and Extracellular Calcium Levels

**DOI:** 10.1101/2025.07.13.664578

**Published:** 2025-09-06

**Authors:** Jade H. Cleary, Kara J. Wentzel, Abigail J. Howard, Adrian M. Sackheim, Sophia H. Piffard, Rushi Sonawane, Mohamed S. Ahmed, Lydia Sheeser, J. Cory Benson, Grant Hennig, Dev Majumdar, Mohamed Trebak, Mark T. Nelson, Kalev Freeman

**Affiliations:** 1 Department of Emergency Medicine, University of Vermont, Burlington VT;; 2 Department of Pharmacology and Chemical Biology and Vascular Medicine Institute, University of Pittsburgh, Pittsburg PA;; 3 Department of Surgery, University of Vermont, Burlington VT;; 4 Department of Pharmacology, University of Vermont, Burlington VT;; 5 Division of Cardiovascular Sciences, University of Manchester, Manchester UK

## Abstract

We tested the hypothesize that the ubiquitous store-operated Ca^2+^ entry (SOCE) contributes to histone-induced endothelial cell Ca^2+^ events. We also considered an alternate hypothesis: cationic electrostatic interactions between histones and negatively charged phospholipids, deforming endothelial cell membranes and thereby allowing extracellular Ca^2+^ entry. A role for SOCE in histone responses was ruled out by genetic ablation of the ORAI1/2/3 channel trio; histone effects were blocked by application of the multivalent cation gadolinium Gd^3+^. Using live cell video microscopy of endothelial cell labeled with membrane dye FM1–43, we recorded fast plasma membrane movements including vesiculation, blebbing, and ruffling of lamellipodia over 60 minutes following histone exposure. These cell membrane theatrics were markedly different from the uniform pattern of rapid exocytosis and subsequent blebbing produced by calcium overload with ionomycin. The membrane permeabilization produced by histones, and not ionomycin, was transient and a subset of cells recovered membrane integrity within 1 hour. Removal of extracellular Ca^2+^ prevented histone-induced intracellular Ca^2+^ overload while surprisingly exacerbating plasma membrane deformation. Conversely, decreasing the density of the negative charge surface by adding calcium or or increasing extracellular Ca^2+^ levels effectively screened common membrane phospholipids from interactions with labeled histones and prevented endothelial damage in cells exposed to histones. Collectively these results indicate that low extracellular Ca^2+^ levels enhance interactions between histones and endothelial cell membrane phospholipids to increase cytotoxicity. Importantly, this supports the concept of aggressive Ca^2+^ repletion during resuscitation to prevent hypocalcemia, stabilize endothelial cell membranes and improve cardiovascular recovery from shock.

## Introduction

Cardiovascular shock secondary to trauma and sepsis is among the most common causes of morbidity and mortality worldwide^1,2^. Over 60 trillion interconnected vascular endothelial cells (ECs) connect all organs and regulate function in the body^3^. The sudden and devastating disruption of endothelial function that occurs locally and in distant organs during acute shock conditions, known as shock-induced endotheliopathy, disrupts the regulation of blood flow, clotting and barrier functions, thereby contributing to progressive organ failure and mortality^4–6^. Despite the emerging consensus that the release of cytotoxic histone proteins into the bloodstream, both from injured cells and activated neutrophils in the form of extracellular traps, is the inciting event that drives acute endothelial injury in trauma and severe infection^7–13^, there are no proven treatments, and the fundamental mechanisms that underlie endothelial cell responses to severe injury remain largely unknown.

The results of clinical trials showing that transfusion of healthy donor plasma to severely injured patients in the early prehospital setting (before arrival to the hospital) decreases biomarkers of endothelial injury and improves survival has transformed emergency care, and clinical protocols used at trauma centers for massive transfusion of blood products are evolving to these changes. Hypocalcemia is a common complication during the resuscitation of critically injured patients, resulting from factors including the infusion of blood products containing sodium citrate (which has an anticoagulant effect by chelating Ca^2+^ ions) and decreased hepatic clearance of the citrate due to impaired perfusion^13^. Low plasma Ca^2+^ levels in trauma are predictive of mortality and need for multiple transfusions^14,15^. Despite the prevalence of hypocalcemia in trauma patients, there is controversy regarding the appropriate thresholds, timing and extent of Ca^2+^ repletion during massive transfusion protocols^16,17^.

We previously demonstrated that histones initiate large endothelial cell Ca^2+^ oscillations that are pro-inflammatory, pro-thrombotic, and promote barrier breakdown, but the ion channels responsible for these Ca^2+^ transients remain unknown^11,18,19^. Store-operated calcium entry (SOCE) is a ubiquitous and ancient signaling pathway present in virtually all cell types that is crucial for numerous cellular functions, including endothelial immune and proliferative responses^20–22^. We therefore hypothesized that SOCE contributes to histone-induced endothelial cell responses. We also considered an alternate hypothesis: cationic histones interact electrostatically with negatively-charged phospholipids on the endothelial cell surface, deforming the plasma membrane and allowing extracellular Ca^2+^ to enter the cytosol. This is also plausible because histones can bind phospolipids and disrupt lipid bilayers^23–25^. If true, then an important corollary to this hypothesis is that low Ca^2+^ would exacerbate endothelial toxicity by increasing the negative charge density of the local electric field near the cell surface, further attracting histones. We studied ECs both in culture and in surgically opened vascular preparations using live cell video microscopy, to address the following questions: (1) Are histone-induced Ca^2+^ oscillations SOCE-dependent? (2) Is Ca^2+^ influx necessary for histone-induced toxicity? (3) Do extracellular Ca^2+^ levels modulate the membrane effects of histones? (4) To what extent are histone-induced membrane changes dependent on the negative charge density of the local electric field near the cell surface?

## Results

### Store operated calcium entry does not contribute to histone-induced endothelial cell calcium events.

**Figure 1.**
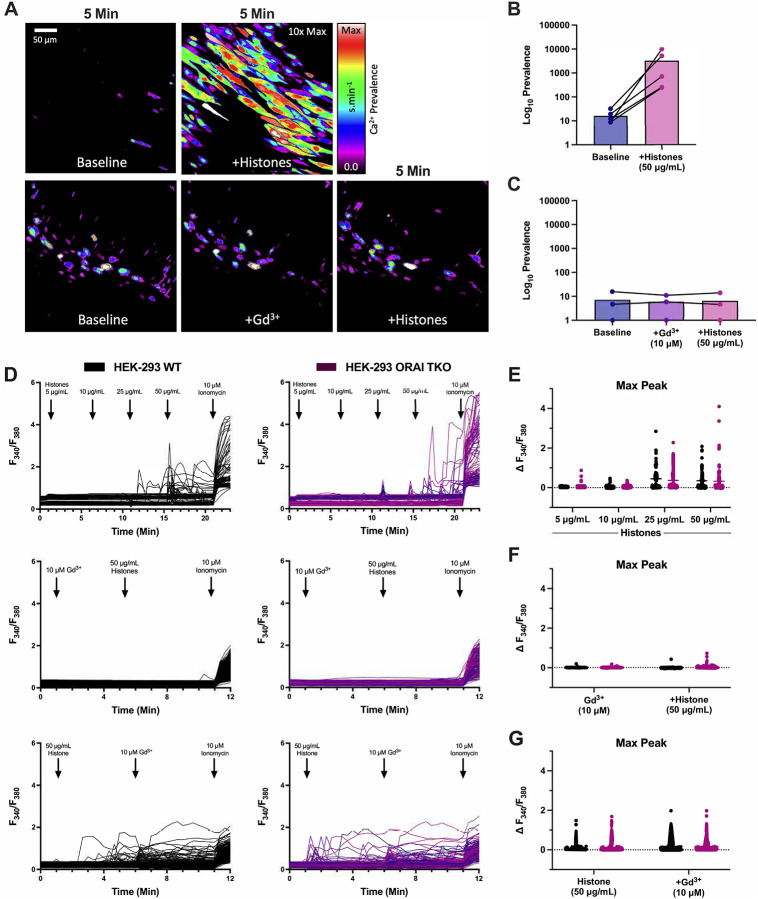
Pre-treatment with Gd^3+^ blocks histone-induced Ca^2+^ signals independent of ORAI channels. (A) En face murine arteries were stained with fluorescent Ca^2+^ indicator (Cal-520, 5 μM) and stimulated with histones (50 μg/mL) with or without Gd^3+^ (10 μM) pre-treatment- Representative, cumulative Ca^2+^ prevalence images from continuous fields of view (FOV) showing surgically-opened murine endothelium at baseline followed by 5 minutes of histone stimulation (top) or after pretreatment withGd^3+^ followed by 5 minutes of histone stimulation. (B) Summary of Ca^2+^ activity in en face cells at baseline compared to histone stimulation. (C) Summary of Ca^2+^ activity at baseline, after Gd^3+^ pre-treatment, and during subsequent histone stimulation. Ca^2+^ signal was quantified as percent of the FOV area with oscillating cells using a specialized standard deviation method, normalized to the ionomycin-induced maximal response. (D) Wildtype (WT, left) and ORAI triple knockout (TKO, right) HEK 293 cells were stained with Ca^2+^ indicator (Fura-2) and stimulated with histones (n=1). Ionomycin (10 μM) was added at the end of each experiment as a positive control. Representative traces of fluorescence over time in response to increasing concentrations of histones (top), pretreatment with Gd^3+^ (10 μM) followed by histones (50 μg/mL) (middle), and Gd^3+^ (10 μM) post-treatment (bottom). Quantification of maximum peak intensity for each cell following histone titration (E), Gd^3+^ pre-treatment (F), and Gd^3+^ post-treatment (G).

To test the hypothesis that SOCE contributes to histone-induced endothelial cell Ca^2+^ elevations, we first employed a pharmacological approach using Gd^3+^, a trivalent lanthanide that is useful when applied at low concentrations (<10 μM) as a SOCE inhibitor^21^. Freshly harvested murine mesenteric arteries, surgically opened to expose the endothelium, were loaded with a fluorescent Ca^2+^ indicator and exposed to clinically relevant concentrations of histones (50 μg/mL) with Ca^2+^ (1.2 mM) in the presence or absence of Gd^3+^ (10 μM) (Figure 1 A–C, Supplemental Videos 1 and 2). Rapid (<5 minutes) and irregular increases in endothelial cell Ca^2+^ fluorescence were observed as expected. These Ca^2+^ oscillations were recapitulated in HEK-293 cells loaded with the ratiometric Ca^2+^ indicator Fura-2 and stimulated with increasing concentrations of histones. Ca^2+^ signals were present in response to histones starting at concentrations of 25 μg/mL, and high amplitude calcium oscillations were observed in response to 50 μg/mL of histones (Figure 1 D,E). As we previously demonstrated in surgical vascular EC preparations^11^, extracellular Ca^2+^ is necessary for histone-induced Ca^2+^ events. When HEK-293s were exposed to histones (50 μg/mL) in a solution of 0 mM Ca^2+^, no Ca^2+^ activity was observed. After 5 minutes, the extracellular solution was replaced with 2 mM Ca^2+^ and a significant increase in activity immediately after the extracellular Ca^2+^ levels were restored. Similar to our findings in ECs, we found that pre-treatment with Gd^3+^ (10 μM) completely blocked histone-induced Ca^2+^ signals in HEK-293s (Figure 1 D,F). Interestingly, post-treatment with Gd^3+^ (10 μM) within 5 minutes of histone (50 μg/mL) exposure was unable to quench histone-induced Ca^2+^ influx (Figure 1 D,G). We additionally tested a lower concentration of Gd^3+^ (5 μM) which should be effective at inhibition of SOCE-dependent Ca^2+^ activity^21^. In contrast to the complete block achieved by 10 μM Gd^3+^, this lower concentration was unable to inhibit histone-induced Ca^2+^ increases in either pre-treatment or post-treatment conditions (Supplemental Figure 1A,B). Since the inhibitory activity of Gd^3+^ for SOCE is in the nM range, with an IC50 of less than 100nM^26^, and 5 μM Gd^3+^ pre-treatment was insufficient to achieve a complete block of histone-induced Ca^2+^ signals (Supplemental Figure 1), these results suggest that SOCE does not contribute to the observed responses.

Therefore, to definitively rule out a role for SOCE, we applied a complementary genetic approach^27^. ORAI channels are key mediators of SOCE through their binding to STIM1, and knocking out all three ORAI homologs completely prevents functional SOCE responses^27–29^. We utilized ORAI triple knockout (TKO) HEK293 cells to determine the role of SOCE in histone-mediated Ca^2+^ signals^21^. There was no difference in histone-induced Ca^2+^ activity between wildtype and ORAI-TKO HEK-293 cells (Figure 1D–G). Again, the addition of Gd^3+^ completely blocked histone-induced Ca^2+^ activity in the ORAI-TKO HEK-293. These results effectively rule out ORAI channels as a contributor to histone-induced Ca^2+^ influx in ECs.

### Histones bind and deform endothelial cell plasma membranes.

**Figure 2.**
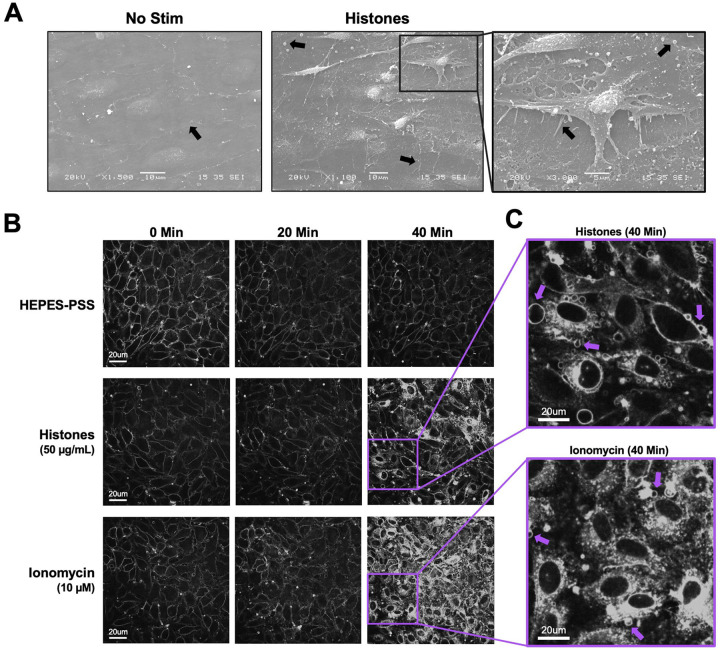
Cultured ECs exhibit morphological changes and FM1–43 dye internalization in response to histones and ionomycin over 40 minutes of exposure. (A) Electron microscopy of native endothelial cells exposed en face in murine blood vessels following exposure to histones (50 μg/mL) with arrows pointing to areas of interest, including potential Weibel Palade bodies, retraction fibers, and blebs or extracellular vesicles. (B) Representative images of cultured cells stained with membrane dye FM1–43 during exposure to buffered saline, histones (50 μg/mL), or ionomycin (10 μM). The same field of view is presented at baseline (0 minutes), 20 minutes, and 40 minutes after exposure. (C) Magnification of histone-stimulated and ionomycin-stimulated cells at 40 minutes, with arrows pointing to examples of bleb formation on cell membranes.

The observation that histones-induced Ca^2+^ entry was independent of ORAI channels, and Gd^3+^ only blocked responses at concentrations greather than 5 μM, suggested the blocking effect of Gd^3+^ must occur through a different mechanism. Multivalent cations such as Gd^3+^ can neutralize the negative charge at the membrane surface, altering the local electric field near the membrane^30,31^. If histones bind to and directly disrupt lipid bilayers, as others have shown^23,24^, then Gd^3+^ at sufficiently high concentrations might screen the cell surface from binding to cationic histones, preventing histone-induced membrane damage or pore formation that would allow entry of Ca^2+^ from the extracellular space^25^. To evaluate the impact of histones on plasma membrane structure and morphology in ECs, we initially performed scanning electron microscopy on *en face* murine mesenteric arteries following stimulation with histones (50 μg/mL) for 30 minutes in HEPES-PSS. Untreated arteries visualized *en face* appeared to have a flat surface with intact cell-to-cell junctions. The plasma membranes appeared smooth and minimal extracellular debris or vesicles were noted (Figure 2A). In contrast, the endothelial surface of the arteries showed significant alterations in cell morphology after treatment with histones. Cells appeared to have retracted, causing a breakdown of cell-to-cell junctions and exposure of retraction fibers. Numerous blebs and extracellular vesicles were also visualized on the endothelial surface of the histone-treated arterial preparations using electron microscopy (Figure 2A).

**Figure 3.**
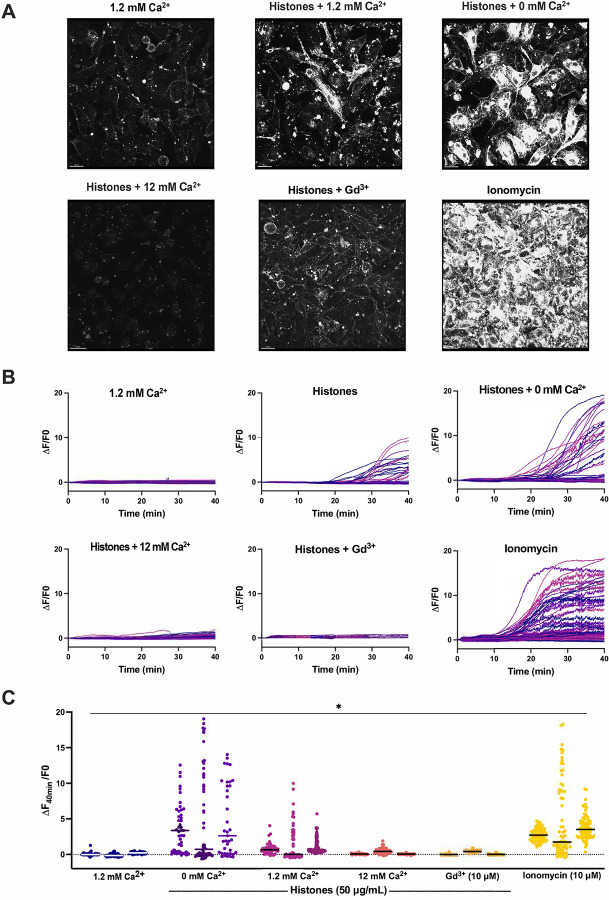
Histone-induced endothelial cell membrane deformation and lysis are exacerbated by low Ca^2+^ and blocked by high Ca^2+^ or Gd^3+^. (A) Representative Z-stacks of cultured ECs after 40-minute exposure to histones (50 μg/mL) under conditions of low (0 mM), normal (1.2 mM), or high (12 mM) Ca^2+^, or with normal Ca^2+^ following Gd^3+^ (10 μM) pre-treatment. (B) Representative traces of individual cell cytoplasmic fluorescence (ΔF/F0) over 40 minutes for each experimental condition, quantified using ImageJ. (C) Final fluorescence (ΔF_40min_/F0) for each cell with replicates displayed separately (n=3) for each condition. *Kruskal-Wallis test; P<0.05.*

These endothelial cell shape changes after histone exposure encouraged us to attempt visualization of plasma membrane responses in live endothelial cells. We used the styryl pyridinium dye FM1–43, which fluoresces when inserted in lipid bilayers, to label the plasma membranes of cultured endothelial cells for live cell imaging. FM1–43 staining can not only reveal changes in cell morphology, but also it can reveal cell membrane disruption and permeability. While normally impermeable to cells, FM1–43 floods the cytoplasm and stains internal membrane structures of damaged cells or those permeabilized by the opening of aqueous pores^32^. We compared the membrane effects of histones to those of ionomycin at a concentration sufficient to mobilize intracellular stores and produce sustained Ca^2+^ influx in cultured ECs^33^. Both stimuli resulted in significant plasma membrane permeabilization over a period of 40 minutes. The endothelial response to ionomycin was characterized by synchronized cytoplasmic filling of the membrane dye between 20 minutes to 30 minutes of exposure, culminating in 92% (± 12%) of cells showing a 2-fold or greater increase in fluorescence at 40 minutes (Figure 3). In comparison, the endothelial response to histones also showed membrane dye internalization with regions of interest defined by cell boundries, but only in a subset of ECs (34 ± 7%) within 40 minutes at normal Ca^2+^ levels (1.2 mM). In the remaining ~ ⅔ of cells, there were either no changes in dye entry, or dye entry was limited to a subcellular region of the cell. The timing of dye entry was also not uniform across cells, with few cells responding to histones before 20 minutes (Figure 3); membrane dye internalization increased between 20 to 40 minutes, much later than the shorter interval (~2–5 minutes) required for histones to produce Ca^2+^ surges (Figure 1).

**Figure 4.**
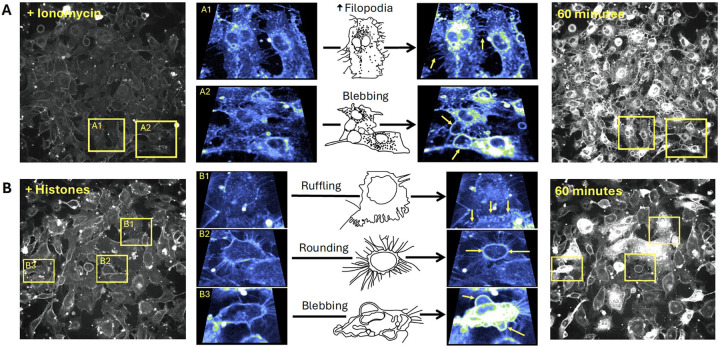
The patterns of plasma membrane movements produced by histones are different than those caused by Ca^2+^ overload with ionomycin. Examples of the distinct patterns of membrane movements observed during video imaging of EC monolayers over a 60-minute time frame are presented as both microscopy images and cartoons, with accompanying video supplements. (A) Ca^2+^ overload with ionomycin caused similar changes in almost all cells across the field of view. The initial response consisted of rapid membrane dye incorporation into the cytoplasm concomitant with the formation of small punctate vesicular bodies located both intracellularly and in the bathing media. Following dye incorporation, numerous filopodia were formed that elongated outwards from the cell body. These filopodia were motile and not attached to the surface of the slide (A1: Supplemental Video 5). The formation of large blebs marked the final response of cells before plasma membrane integrity was irrevocably lost (A2: Supplemental Video 6). (B) Histone exposure produced a heterogenous, mosaic pattern in which some cells respond rapidly and dramatically while others appear largely unaffected (Supplemental Video 7). Dye uptake occurred in a patchy fashion in cells, often initiating at one or more regions before spreading throughout the cell body. Extracellular vesicles appear to be both released and reabsorbed. Unique to the histone response, membrane ruffling behavior was observed at the edges of some cells, with undulating movements and expansion of the membrane border away from the cell body (B1: Supplemental Video 8) whilst other cells underwent rapid retraction of the outer membrane (rounding), leaving behind retraction fibers that remain connected to the media surface and adjacent cells (B2: Supplemental Video 9). A subset of cells showed similar end-stage membrane movements with large amounts of blebbing and dye uptake (B3: Supplemental Video 10). FOV Width = 490μm. Sub panel width = 70 – 97 μm.

Close inspection of the video recordings demonstrated active membrane deformations^34^ and release of extracellular vesicles over the 60 minute recording period, in response to both ionomycin and histone, but the extent and pattern of plasma membrane responses to these stimuli were qualitatively different (Supplemental Videos 3 and 4). To illustrate the patterns of dynamic membrane changes, we present two examples of fields of endothelial cells recorded over 1 hour with zoomed-in regions of interest in order to demonstrate and define the fast plasma membrane movements observed in endothelial cells. (Figure 4). The patterns of membrane movements caused by ionomycin included extension of filopodia and blebbing (Figure 4A, Supplemental Videos 5, 6). Histone exposure resulted in dynamic cell responses in a different pattern. Membrane movements produced by histones and not ionomycin included cell rounding with exposure of retraction fibers and ruffling sheets of plasma membrane, that appear to be ruffling lamellipodium^35^ at the edges of cells particularly at surfaces near adjacent cells (Figure 4B, Supplemental Videos 7, 8). Histone exposure also resulted in an end-stage pattern of blebbing and cessation of cell movements in some cells (Figure 4B, Supplemental Videos 9). Additional examples of histone-induced plasma membrane ruffling and blebbing are also provided (Supplemental Videos 10, 11).

Both histones and ionomycin exposure caused the release of extracellular vesicles. We measured the concentration of Evs <500 um in size in the supernatant after agonist exposure. Compared to buffer alone, the number of particles per mL increased after 60 minutes exposure to either agonists (range for duplicate measurments with buffer alone 2.6 to 4.0 E+7, histones 4.2 to 5.2 E+9, ionomycin 8.6 to 11 E+9 particles / mL). In cells after histone exposure, the rounding of the outer surface of the cell and the release of fragments of apoptotic bodies even larger than 500um could be observed (Supplemental Video 4). While not all cells exposed to histones appeared to release EVs, the response to ionomycin was more uniform, with near-synchronous appearance of punctate, stippled, rapidly vibrating symmetrical membrane particles <5 μm in the early part (<10 minutes) of the 60 minute videos. In some cases, extracellular vesicles were visualized being released by cells, and then absorbed by both adjacent and remote cells. The vesicles even passed between cells directly across connected lamellipodia or filopodia fingers (Supplemental Video 12). This appearance is similar to that described as “exosome surfing”, with single vesicles recruited by filopodia, which grab and then pull the vesicles towards endocytic hot spots at the base of the filopodia on the cell body^36^. We also noted that dye uptake originated from subcellular regions within a single cell then spreads to the rest of the cytoplasm. After initial dye uptake in these “hot spots”, FM1–43 staining near spreads from the outer membrane to internal membrane components, through a process that appears consistent with endocytotic trafficking, until the cell cytoplasm is filled with dye while the nucleus remains unstained.

### Plasma membrane changes are not due to calcium overload.

Based upon the earlier and more rapid occurrence of histone-induced endothelial cell Ca^2+^ influx than plasma membrane changes, we considered the possibility that Ca^2+^ overload was the trigger for subsequent membrane disruption. We previously established extracellular Ca^2+^ is required for histone-induced Ca^2+^ entry into vascular endothelial cells^11^. We reasoned that, if histone-induced endothelial cell Ca^2+^ overload drives cell membrane changes, the removal of extracellular Ca^2+^ should prevent these membrane responses. We therefore tested the impact of low Ca^2+^ on histone-induced plasma membrane responses visualized with FM1–43 in endothelial cells cultured to confluence (60 ± 17 cells per field of view). Surprisingly, exposure to histones in low Ca^2+^ exhibited an exaggerated response compared to normal extracellular Ca^2+^ conditions (1.2 mM). While histone-exposure caused significant dye uptake in 34% (± 7%) of cells at 1.2 mM Ca^2+^ levels, this value increased to 60% (± 11%) of cells at 0 mM Ca^2+^ concentrations. Similarly, the average highest change in fluorescence at 40 minutes increased from F/F0 of 6.56 at 1.2 mM Ca^2+^ to 18.7 at 0 mM Ca^2+^ (Figure 4). Qualitatively, the patterns of cell membrane deformation, including blebbing and cell produced by histones under low Ca^2+^, appeared more extensive than that produced in normal Ca^2+^ containing solutions (Figure 4). Using electric cell-substrate impedance sensing (ECIS), we also also tested the effects of extracellular Ca^2+^ on endothelial barrier function of endothelial cells. In buffered saline with physiological Ca^2+^ levels (1.2 mM), treatment with histones (100 μg/mL) activated endothelial cells over a one hour period, increasing the normalized endpoint resistance and total resistance of the barrier to electrical currents under normal buffer conditions (Supplemental Figure 3A). In contrast, histones in low Ca^2+^ buffer produced in an nearly immediate and signficant decrease in both normalized endpoint resistance (0.5 ± 0 Ω n=7 vs 1.7 ± 0.1 Ω n=8) and total resistance (AUC 462 ± 9 Ω n=7 vs 1558 ± 81 Ω n=8) beginning within 10 minutes of exposure (Supplemental Figure 3B and C).

### Neutralizing cell membrane surface charge prevents histone interactions.

We then investigated the impact of high Ca^2+^ and Gd^3+^ on histone-induced membrane disruption in live ECs. Both high Ca^2+^ (12 mM) and Gd^3+^ (10 μM) were protective. The extent of membrane dye internalization produced by histones was markedly diminished in the presence of high Ca^2+^ or following pretreatment with Gd^3+^, as shown by quantitative spatiotemporal analysis of F/F0 in the video recordings, summarized in Figure 3.

**Figure 5.**
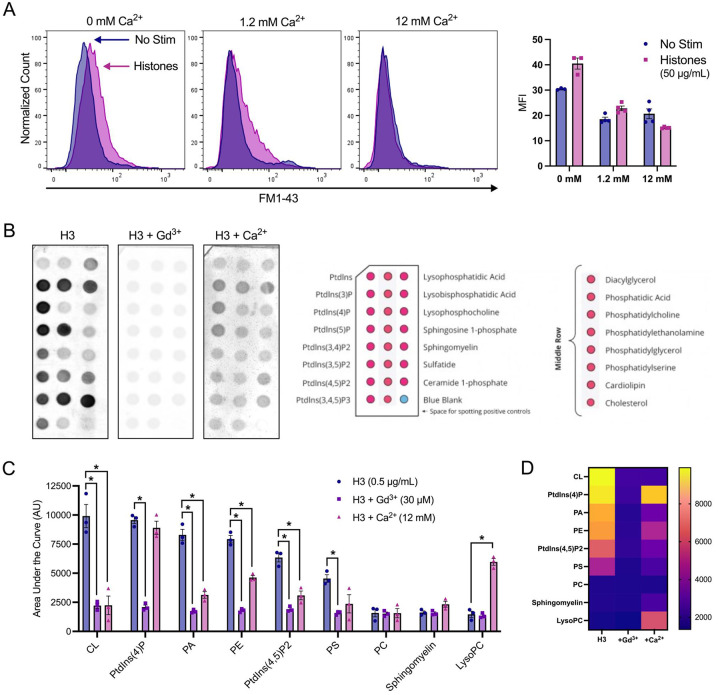
Histone-induced membrane permeabilization and membrane lipid binding can be modulated by extracellular cation concentrations. (A) Flow cytometry of histones on ECs shows exacerbation of membrane permeabilization by low Ca^2+^ (0 mM) and block by high Ca^2+^ (12 mM). Representative flow cytometry histograms showing FM1–43 fluorescence (PE) following incubation of EA.hy926 cells with FM1–43 dye and 50 μg/mL histones or 10 μM ionomycin for 40 minutes in buffered saline solution (HEPES-PSS) with zero added Ca^2+^ (0 mM), normal Ca^2+^ levels (1.2 mM), or high Ca^2+^ (12 mM). The mean fluorescent intensity (MFI) was quantified using the median fluorescence for cells exposed to 50 μg/mL histones at different Ca^2+^ concentrations. *Kruskal-Wallis test; P<0.05. (B)* Histone H3 selectively binds to membrane bound lipids in a charge dependent manner. Representative images of lipid binding strips exposed to histone H3 alone, in the presence of Gd^3+^ (30 μM), or in the presence of Ca^2+^ (12 mM) (left). Schematic diagram of lipid identities, adapted from the manufacturer (Right; Echelon Biosciences, Alabama, USA). (C) Summary data for histone H3 binding to cardiolipin, Phosphatidylinositol 4-phosphate (PtdIns(4)P), phosphatidic acid (PA), phosphatidylethanolamine (PE), Phosphatidylinositol 4,5-bisphosphate (PtdIns(4,5)P2), and phosphatidylserine (PS). phosphatidylcholine (PC), sphingomyelin, and lysophosphocholine alone, or in the presence of Gd^3+^ (30 μM), or Ca^2+^ (12 mM). Binding was quantified in ImageJ as Area Under the Curve (AUC). N=3 for each group. *Two-Way ANOVA with Bonferroni’s Correction for Multiple Comparisons; P<0.05*.

To confirm our microscopy findings, we used flow cytometry to quantify endothelial cell FM1–43 update after histone exposure under conditions of normal or high Ca^2+^ compared to Ca^2+^ free conditions. Flow cytometry results recapitulated the pronounced internalization of FM1–43 in a subset of cells following exposure to histones (50 μg/mL) in both normal and low Ca^2+^ conditions. The mean fluorescent intensity (MFI) of FM1–43 increased 1.23-fold following histone exposure in 1.2 mM Ca^2+^ and 1.33-fold in 0 mM Ca^2+^. Conversely, cells stimulated with histones in 12 mM Ca^2+^ showed a 1.36-fold decrease in MFI relative to unstimulated cells at the same Ca^2+^ concentration (Figure 5A). These findings further support that histone interactions with endothelial cell membranes can be modulated by extracellular Ca^2+^ concentrations.

The pronounced effects of extracellular Ca^2+^ and Gd^3+^ on blocking histone cytotoxicity indicated that histone-induced membrane effects depend on strength of the plasma membrane surface charge and occur independently of SOCE and Ca^2+^ entry. Thus, we hypothesized that these cations protect ECs from histones by directly neutralizing negatively-charged phospholipids and reducing electrostatic interactions between histones and the plasma membrane. To test this, we measured the binding affinity of a tagged-histone protein (GST-H3) to individual phospholipids found commonly in biological membranes in the presence or absence of elevated Ca^2+^ or Gd^3+^. Like prior studies^11^, we observed that in the absence of Ca^2+^, histones strongly bind to anionic not zwitterionic membrane phospholipids, consistent with an electrostatic interaction (Figure 5B,C). We found that Gd^3+^ (30 μM) completely blocked all histone-phospholipid interactions, removing the preferential binding motifs. Similarly, high Ca^2+^ (12 mM) significantly reduced histone binding to most phospholipids.

### Phosphatidyl serine translocation to outer leaflet occurs in response to histone stimulation even in the absence of external calcium.

Histone stimulation of endothelial cells promotes thrombosis in part due to translocation of phosphatidyl serine (PS) to the membrane surface through a process that is inhibited by blocking the calcium-activated phospholipid scramblase TMEM16F^28^. However, increased PS on the external leaflet of the plasma membrane may also indicate programmed cell death, so it is not clear if the Ca^2+^ overload produced by histones activated the scramblase directly to produce transient changes in PS or if the cells were pre-apoptotic and destined for cell death. We were therefore interested to determine whether histone-exposed PS translocation can be modulated by extracellular Ca^2+^ concentrations, and the extent to which these cells also permeable to to the viability dye propidium iodide (PI). Following 1 hour of histone exposure in HEPES-PSS containing varying concentrations of Ca^2+^ (0, 1.2, 12 mM), cells were stained with Annexin V for PS exposure and PI for permeability then evaluated by flow cytometry. At a moderate histone dose of 50 μg/mL, cells at normal Ca^2+^ levels of 1.2 mM showed a 5-fold increase in PS^+^/PI^−^ cells to 3.85% (± 0.22%) relative to unstimulated cells at the same Ca^2+^ level. This percentage dropped to 2.73% (± 0.55%) at 0 mM Ca^2+^ and 1.16% (± 0.33%) at 12 mM Ca^2+^, which was almost identical to unstimulated cells at all Ca^2+^ levels (Supplemental Figure 2). Similarly, 50 μg/mL histones resulted in 8.40% (± 1.69%) of cells that were double positive (PS^+^/PI^+^) at 1.2 mM Ca^2+^ which represents a 2-fold increase relative to unstimulated cells. Like the PS^+^/PI^−^ findings, this percentage dropped to 6.19% (± 2.97%) at 0 mM Ca^2+^ concentrations. Notably, unstimulated cells in the 12 mM Ca^2+^ condition showed an elevated PS^+^/PI^+^ percentage at 8.18% (± 0.28), matching the respective histone-stimulated population of 8.29% (± 2.76%). Thus, high Ca^2+^ levels were protective from histone-induced cytotoxicity, but caused increase PS exposure and PI uptake at baseline (Supplemental Figure 2).

Since 50 μg/mL of histones did not cause dramatic changes in PS exposure or PI uptake, we next tested a higher dose of 100 μg/mL histones which we have previously shown was sufficient to produce PS translocation in endothelial cells^37^. At this higher dose, 32.15% (± 7.37%) of cells were PS^+^/PI^−^ at 1.2 mM Ca^2+^ which dropped to 24.20% (± 4.95%) at 0 mM Ca^2+^ and 3.52% (± 1.32%) at 12 mM Ca^2+^. When compared to unstimulated controls at the various Ca^2+^ levels, these changes represent 16-fold, 14-fold, and 1.5-fold increases in PS^+^/PI^−^ percentages for 1.2 mM Ca^2+^, 0 mM Ca^2+^, and 12 mM levels, respectively. Similarly, the PS^+^/PI^+^ percentages were 13.38% (± 3.02%), 12.93% (± 1.82%), and 4.11% (± 1.05%) following 1 hour of 100 μg/mL histone stimulation at 12 mM, 0 mM, and 12 mM Ca^2+^ concentrations, respectively. Interestingly, since the percentage of PS^+^/PI^+^ cells in the unstimulated 12 mM Ca^2+^ condition was elevated from 5.51% (± 1.01%) in normal Ca^2+^ to 8.14% (± 4.13%), the histon-estimulated cells at the 12 mM Ca^2+^ level appeared to have less cell death than their respective control. These results indicate that both moderate and high doses of histones lead to both PS exposure and PI uptake in a subset of cells.

### Membrane permeabilization produced by histones is reversible.

**Figure 7.**
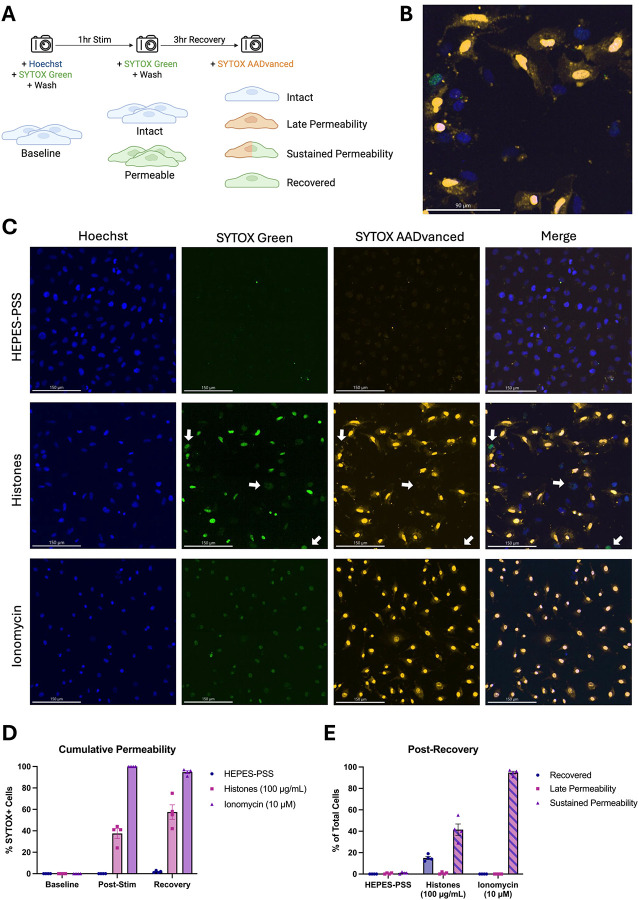
A subset of ECs with histone-induced membrane permeability can recover their membrane integrity after 3 hours. (A) Schematic diagram of experimental design allowing the determination of four cell phenotypes following histone (100 μg/mL) or ionomycin (10 μM) stimulation: 1. Intact (Hoeschst^+^), 2. Late Permeability (Hoeschst^+^ SYTOX-AADvanced^+^), 3. Sustained Permeability (Hoeschst^+^ SYTOX-AADvanced^+^ SYTOX Green^+^), and 4. Recovered (Hoeschst^+^ SYTOX Green^+^). (B) Magnification of one representative field of view showing multiple permeability phenotypes following histone stimulation for 1 hour and recovery for 3 hours. (C) Representative images from the post-recovery timepoint for HEPES-PSS, histones, and ionomycin. Individual fluorescence channels are shown for Hoeschst, SYTOX Green, and SYTOX AADvanced followed by a merge of all channels. (D) Cumulative percentages of cells permeable to either SYTOX dye (SYTOX Green and/or SYTOX AADvanced) at baseline, post-stimulation, and post-recovery for each treatment condition. (E) Percent of cells in each phenotypic category at the post-recovery timepoint following stimulation with HEPES-PSS alone, histones, or ionomycin.

Although these findings show that high doses of histones can kill ECs, we also considered the possibility that a subset of PS-flipped and permeabilized cells could recover their membrane integrity. Hence, we next sought to evaluate cell recovery following histone-induced injury using a novel strategy involving live-cell imaging with different colored viability dyes. Live ECs were initially stained with Hoechst nuclear dye and SYTOX Green to determine baseline viability. The adherent monolayers were then washed multiple times to remove unbound dye and subsequently incubated in HEPES-PSS containing no stimulant, histones (100 μg/mL), or ionomycin (10 μM). Following 1 hour of stimulation, the cells were stained and imaged with SYTOX Green again to determine post-stimulation viability. Finally, the cells were recovered in fresh DMEM with 10% FBS at 37 °C and 5% CO_2_ for 3 hours then stained with red SYTOX AADvanced for post-recovery viability (Figure 7A). Through this experimental design, we were able to use microscope filters to distinguish between 4 distinct cell phenotypes: 1. Intact (blue alone), 2. Late Permeability (blue and red), 3. Sustained Permeability (blue, red, and green), and 4. Recovered (blue and green).

In the histone-treated group, a subset of cells exhibited early membrane permeability to SYTOX Green, but excluded the red SYTOX AADvanced, indicating regained membrane integrity (Figure 7C,E). In contrast, cells treated with ionomycin, a positive control, demonstrated sustained and uniform permeability to both SYTOX Green and red SYTOX AADvanced, consistent with irreversible membrane damage. Figure 7D shows cumulative permeability over time, based on the combined percentage of cells positive for either dye at each timepoint. The increase in permeability observed in the histone group may be partially influenced by variability in imaging fields across timepoints. Figure 7E quantifies the distribution of cells in each condition according to dye uptake classification (as outlined in Figure 7A): SYTOX Green only (“recovered”), SYTOX Green and red SYTOX AADvanced double-positive (“sustained permeability”), or red SYTOX AADvanced only (not observed). At the final timepoint, 15.0% (± 2.9%) of histone-treated cells were SYTOX Green-positive only, indicating recovery. Conversely, 41.5% (± 10.7%) were double-positive, indicating sustained permeability. In the ionomycin-treated condition, 94.8% (± 2.6%) of cells were double-positive, and 0% (± 0%) of cells had recovered membrane integrity. These results indicate that endothelial cell membrane permeabilization is not irreversible; a subset of permeabilized endothelial cells were able to repair their plasma membranes.

## Discussion

The identification of endotheliopathy as a driver of coagulopathy and poor outcomes in shock states of sepsis (e.g., bacterial infection, COVID-19) and trauma (e.g., hemorrhage, brain or multisystem injury, burns)^9,38,39^ was an important advance in critical care medicine. The paradigm of SHINE provides a unifying pathophysiologic mechanism that explains how different shock states converge to produce similar patterns of endothelial activation and injury^4,5^. In this study, we investigated mechanisms of histone-induced endotheliopathy that demonstrate the importance of extracellular Ca^2+^ in shielding electronegative endothelial cell surfaces from histone cytotoxicity. We have previously demonstrated that histones initiate large EC Ca^2+^ oscillations that are pro-inflammatory, pro-thrombotic, and promote barrier breakdown^11,18,19^. Store-operated calcium entry (SOCE) is the most ubiquitous Ca^2+^ entry in non-excitable cells, such as ECs, that is triggered in response to a variety of stimuli such as vascular endothelial growth factor (VEGF) and thrombin^20^. This led to our initial hypothesis that SOCE contributes to histone-induced Ca^2+^ influx; however, our experimental results did not support this model. We then considered an alternate hypothesis: histones may induce Ca^2+^ entry by binding and deforming EC membranes, independently of SOCE. In addition, we aimed to understand the extent to which intracellular Ca^2+^ overload drives histone toxicity^40–42^. We also considered the possibility that removal of Ca^2+^ would increase exposure of negatively charged phospholipids on EC membranes to interact with cationic histones while surface charge screening with the addition of Ca^2+^ or multivalent cations would decrease this interaction. We report 4 novel findings: 1) Activation of plasma membrane SOCE channels ORAI1/2/3 either directly or indirectly through release of intracellular Ca^2+^ stores does *not* contribute to histone-induced cytosolic Ca^2+^ influx; 2) histones bind to and deform ECs, producing fast irregular plasma membrane Ca^2+^ flickers or spikes in a pattern distinctly different from the regenerative Ca^2+^ oscillations induced by physiological agonists^27^ or the large plateaus caused by ionomycin-induced Ca^2+^ influx; 3) Ca^2+^ influx is *not* required for histone-induced endothelial cell deformation, and 4) histone-induced membrane permeability depends on electrostatic interactions with negatively charged membrane phospholipids which are exacerbated by low Ca^2+^ and blocked by mM Ca^2+^ or μM Gd^3+^. These findings provide important insights into the nature of SHINE and highlight the potential cardiovascular risk associated with hypocalcemia during resuscitation.

The release of intracellular Ca^2+^ stores and subsequent activation of ORAI channels is an important mechanism for endothelial and immune cell signaling. Our initial experiments which showed that Gd^3+^ blocked histone-induced endothelial cell Ca^2+^ influx (Figure 1) suggested SOCE may also play a role in histone responses, as Gd^3+^ is a known ORAI channel antagonist^26^. However, we only achieved a block with concentrations that were an order of magnitude beyond those required for inhibit SOCE, and the ablation of all three SOCE channels (ORAI1, ORAI2, and ORAI3) had no impact on histone-induced endothelial cell Ca^2+^ overload (Figure 2). This effectively ruled out SOCE as a mechanism of histone-induced Ca^2+^ signaling. Instead, these observations provided support for an alternate model in which histones allow direct entry of extracellular Ca^2+^ by creating pores and deforming the outer membrane. We visualized the time course and extent of endothelial cell membrane deformation produced by histone exposure at high spatial and temporal resolution for the first time using video imaging of endothelial cell plasma membranes labeled with the fluorescent dye FM1–43. This dye has been used to study endothelial cell membrane injury produced by other insults such as laser injury^32,43^, but we are not aware of prior studies using this dye to study histone responses. We found that, indeed, histones deform endothelial cell membranes and produce a large increase in membrane fluorescence within cells. Membrane dye uptake after histone exposure was statistically significant, measured both by spatiotemporal analysis of video results and by separate flow cytometry experiments.

Utilizing live cell confocal microscopy with the membrane label FM1–43, we visualized suprisingly dynamic membrane responses to histones (Figure 4). Histones produced fast plasma membrane movements that included examples of 1) lamellipodia projection and ruffling, 2) release of cell membrane fragments, 3) retractions of lamellipodia, 4) blebbing of the cell surface and 5) cellular collapse with rapid membrane dye internalization. The heterogeneity in response was also notable with only a subset of cells in each experiment responding to histones while other cells appeared unaffected. Moreover, the pattern of membrane response even at the subcellular level was not uniform, with membrane changes initially isolated to “hot spots”, which initially spread across the membrane surface of the cell and then eventually resulted in the entire cell filling with dye. In some examples, extracellular vesicles were observed floating to the cell surface and this contact appeared to initiate the subcellular spreading events. This imaging pattern is consistent with endocytocytic trafficing of the labeled lipidsIn the remaining cells, there were either no changes in cell permeability or dye entry was sequestered to hot spots within cell protrusions without progressing further. The heterogeneity in endothelial cell responses within a given field of view is consistent with other studies of both cultured cells and native endothelium, in which only a fraction of cells showed Ca^2+^ entry or PI uptake after histone exposure^11^. Endothelial cell heterogeneity, even within a single blood vessel, has also been recognized in other systems; for example, only a subset of aortic ECs respond to laminar flow^44^. It is possible that distinct subpopulations of ECs, even within a field of cultured cells, serve as sentinal cells with enhanced susciptibilty to mechanical or chemical insults, acting to amplify the danger signal and activate surrounding cells. The non-sentinal cells in the same field might respond to activating signals by the protrusion and ruffling of lamellipodia in thin sheets that can provide a temporary increase in barrier function. This would explain the initial increase in barrier function measured by ECIS in response to histone activation that we (Supplemental Figure 3) and others have observed in the first hour of exposure to histones, which preceeds eventual degrdation of barrier function that results over longer time periods (12–24 hours)^45,46^.

We were also surprised to find that Ca^2+^ is not required for histone-induced endothelial cell deformation. Intracellular Ca^2+^ overload produced by any means, whether by SOCE or disruption of membrane integrity, can destabilize cell membranes and lead to activation of cell death pathways^47^. The peak timing of the histone-induced membrane changes (30–40 minutes) was later than when we observed the Ca^2+^ surges (3–5 minutes). However, exposure to ionomycin, which elevates Ca^2+^ to a maximum level, produced a different pattern of membrane activation from that of histones. Cells that are activated by ionomycin did not show extensive projection and ruffling of lamellipodia that we observed with histone exposure. Rather, the endothelial response to ionomycin was characterized by a dramatic, synchronous release of symmetrical (~5 μm diameter) membrane fragments across all cells. This pattern appears consistent with exocytosis of preformed vesicles which likely represented Ca^2+^ dependent exocytosis of Weibel-Palade bodies^48,49^. We also visualized extracellular vesicles being re-absorbed by both adjacent and remote cells; extracellular vesicles were also passed between cells directly across connected lamellipodia (Supplemental Video 12).

Surprisingly, exposure to histones in low Ca^2+^ exhibited an exaggerated response compared to normal Ca^2+^ conditions (Figure 5). The patterns of dye entry and cellular deformation produced by histones under low Ca^2+^ conditions matched those of normal Ca^2+^ but they were more extensive. The extent of membrane thickening, blebbing, release of membrane fragments appeared visibly more extensive. This was unexpected, because removal of extracellular Ca^2+^ prevents histone-induced endothelial cell Ca^2+^ events^11^ so it seemed plausible that Ca^2+^ overload would contribute to membrane effects. However, these results were additionally confirmed by flow cytometry using FM1–43. Using ECIS, we also noted that barrier function was rapidly disrupted by histones under low Ca^2+^ but not normal Ca^2+^ conditions (Supplemental Figure 3). Thus, low extracellular Ca^2+^ concentrations make ECs more susceptible to histone-mediated membrane permeability.

In contrast, we also observed that elevated levels of extracellular Ca^2+^ could entirely block membrane permeability in ECs. We also found that both high Ca^2+^ or Gd^3+^ blocked binding of GST-tagged histone H3 to various membrane lipids, indicating that these compounds can interfere with histones binding to the plasma membrane. Interestingly, only μM concentrations of Gd^3+^ were required to completely block membrane permeability following histone exposure. In comparison, there was not a substantial block observed by 1.2 mM Ca^2+^ levels which indicates the vast difference in neutralizing ability between these two compounds. While both are highly cationic, Gd^3+^ binds more readily and strongly due to its higher charge and greater charge density. These results support a model in which cationic histones bind the negatively charged phospholipids in the cell surface through electrostatic interactions exacerbated when during hypocalcemia due to loss of surface charge screening by calcium.

Finally, we found that high doses of histones lead to exposure of PS and uptake of viability dyes after 1 hour. These findings align with prior literature on the endothelial cell toxicity of histones and their ability to increase PS and activate pro-coagulatory changes^7,8,11,25,37^. Most notably, the exposure of PS not only indicates endothelial activation, but could also represent a positive feedback loop in which histones are more readily able to bind to the membranes of activated or pre-apoptotic cells. Since PS is a highly negatively charged phospholipid that avidly binds histones (Figure 5), its presence on the outer leaflet of the plasma membrane may cause pro-coagulatory or pre-apoptotic cells to become even more highly susceptible to histone binding.

In addition to these findings, we also present novel evidence that a subset of endothelial cells can recover from histone exposure. Although only a subset of histone-treated cells appeared to recover membrane integrity (~15%) after 3 hours, this population is notable given that ionomycin-treated cells consistently showed 0% recovery across all trials. These observations point to the existence of functionally distinct subpopulations within the histone-treated group. It seems that histones may induce severe, irreversible damage in a subset of highly susceptible cells while others experience a more moderate, transient injury that permits membrane repair. Interestingly, a similar mosaic pattern of endothelial cell calcium responses within a monolayer was reported in response to shear stress supporting the emerging concept that a distinct subpopulation of cells within endothelial monolayers serve as sentinals that detect and respond to local environmental cues^44^.

In conclusion, our studies demonstrate that low Ca^2+^ levels enhance the interactions between histones and endothelial cell membranes, leading to increased cytotoxicity. Conversely, elevated Ca^2+^ levels – or treatment with agents that alter provide surface charge screening effects such as Gd^3+^ – block histone-phospholipid interactions and mitigate endothelial damage. The effects of Ca^2+^ and Gd^3+^, and lack of effect with knocking out ORAI channels, support a model in which histone binding to cells occurs primarily due to electrostatic interactions with negatively charged membranes.

Intravenous Ca^2+^ salts are used clinically, including during cardiac resuscitation and in hyperkalemia. The stabilizing effects of Ca^2+^ on nerve and cardiac muscle cell membranes are usually explained by the surface charge theory, in which higher Ca^2+^ levels decrease the density of the negative charge surface near the sodium channel of myelinated nerve fibers, making it harder for sodium ions to enter the cell and trigger an action potential^30,31^. For all cell types, the plasma membrane surface itself is more electronegative than the lipid surface of cellular organelles, as shown by measurement of the binding of prenylated cation probes^50^. That is because electric fields generated by cells depend not only on lipids but also on ionizable components embedded in the external membrane surface. In vascular endothelial cells, sugar moieties of the specialized glycocalyx confer additional net negative charge to the outer surface^51^. This is critical to physiologic regulation of blood clotting, because the strong electronegative field of the inner surface of blood vessels serves to repel the negatively charged circulating platelets and blood components. In fact, early experiments showed that reversal of transmural potential across the aorta produced gradual intravascular thrombus produces in complete aortic occlusion within 9 days, and resulting biophysical models of charge characteristics were important in the development of vascular grafts and surgical procedures^52,53^. However, the electric field generated by the net negative surface charge of the vascular endothelium also serves to recruit extrinsic cationic proteins such as cytotoxic histones. We show here that changes in the endothelial cell surface charge produced by elevated Ca^2+^ or by the addition of Gd^3+^ to an extent sufficient to decrease the density of negative charge surface can screen membrane phsopholipids from attracting cytotoxic histone proteins. This conclusion is supported by prior findings on membrane dynamics and the mechanisms of histone cytotoxicity. For example, some proteins can bind to the curved surface of cell membranes and thereby bend them, with resulting membrane deformations that indirectly attract each other, producing membrane invaginations that lead to vesiculation^54^. This phenomenon may partially explain the mode of action of histones. It is well known that histones are highly cationic proteins that bind negatively charged phospholipids in bilayers and cell membranes^24,25,55–57^, and direct interactions between histones and cell membranes have been implicated in their toxic effects^8,58^. Recently, the globular domain of histones alone was shown to deform and lyse the lipid membranes of liposomes and cultured ECs

While the evidence strongly supports a model in which histone interacts with lipid bilayers directly, independent of protein factors, the differences in histone responses among various cell types has not been explained. For example, neurons are relatively insensitive to histones, compared to macrophages or ECs^59^. Extracellular histones also have other important roles in innate immune responses as components of neutrophil-extracellular traps, for example, which serve to tether neutrophils to vascular walls by binding von Willebrand factor^10,60^. Additionally, it has been shown that downstream immune or pyroptotic responses can vary due to histone post-translational modifications, composition, DNA content, and the cell type^8,55,58,61–63^. Prior literature has also implicated toll-like receptors 2 and 4 (TLR2 and TLR4), the purinergic receptor P2×7, the receptor for advanced glycosylation end-products (RAGE), and/or direct interaction between histones and membrane phospholipids^11,25,46,55,58,59,61,64–70^. It seems likely that histones activate cells through multiple pro-inflammatory mechanisms that lead to coordiated cellular signals which ultimately orchestrate cell responses. Additional research is needed to understand why some ECs appear to function as sentinal cells that are highly susceptible to histones while others can withstand or even recover from exposure.

Our findings have clinical relevance to coagulopathy and endotheliopathy in shock and trauma. Hypocalcemia occurs frequently during trauma resuscitation due to transfusion of citrated blood products^14,15^, but clinical protocols for massive transfusions diverge, with some recommending routine Ca^2+^ administration while others recommend supplementation only as needed^16,17^. Moreover, it is important to consider that, while we focused on ECs, other vascular cells are also sensitive to histones^68,58,62,64^. Future studies will need to evaluate whether the previously reported histone toxicity to vascular smooth muscle cells, erythrocytes, and platelets may also be increased in the presence of low Ca^2+^ conditions^68,69,69^; this might have important implications in the context of resuscitation and massive transfusion protocols. In sum, our results suggest that one of the dangers of untreated chelation-induced hypocalcemia is its potential to amplify histone-induced endothelial injury, underscoring the importance of early and aggressive Ca^2+^ correction during resuscitation. The implementation of standardized protocols to optimize Ca^2+^ administration in critically injured patients has the potential to reduce the incidence of endotheliopathy and improve outcomes.

## Materials and Methods

### Animal husbandry.

Mice were kept on a 12 h light/dark cycle with ad libitum access to food and water and were housed in groups of five. All studies were conducted in accordance with the Guidelines for the Care and Use of Laboratory Animals (National Institute of Health) and approved by the Institutional Animal Care and Use Committee of the University of Vermont.

### Ca^2+^ imaging in blood vessels.

En face murine vascular endothelial preparations were utilized for imaging as previously described^11,19^. Briefly, adult male wildtype C57BL6j (C57) mice (Jackson Laboratories) were euthanized using 5% Isoflurane, 95% O2, and decapitated. The mesentery was immediately harvested and placed in 4°C HEPES-buffered physiological saline solution (HEPES-PSS: 10 mM HEPES, 134 mM NaCl, 6 mM KCl, 1 mM MgCl_2_, 2 mM CaCl_2_, 7 mM glucose, pH 7.4). Mesenteric arteries (100–150 μm internal diameter) were excised in sections of approximately 500 μm, cut longitudinally to expose the lumen, and pinned flat with the endothelial surface exposed en face for imaging. Vessels were loaded with green-fluorescent Ca^2+^ indicator (5 μM Cal-520 AM, Thermo Fisher Scientific) and incubated at 37 °C for 30 minutes prior to imaging

### Scanning Electron Microscopy.

Third order mesenteric arteries (~200 μm diameter) from male C57BL6/j mice were cut open on one side longitudinally (“en face”) and pinned down flat on a small silicone dish to expose the intact endothelium. Arteries were incubated with 50 μg/mL of unfractionated histones (Sigma-Aldrich, USA) for 30 minutes in HEPES-PSS (pH 7.4) at 37 °C. The arteries were then fixed in paraformaldehyde (2%)-glutaraldehyde (2.5%) cacodylate buffer solution (0.1 M; pH 7.2) for 24 h, washed with cacodylate buffer, post-fixed in a solution of 2.5% potassium ferrocyanide for 1 h, and washed again in cacodylate buffer and ultrapure water. Sections were dehydrated in ascending grades of ethanol, subjected to critical point drying in CO_2_ (Samdri-PVT-3D, Tousimis, Rockville MD), coated with 10 nm of pure gold in a vacuum sputter coater (Quorum EMS150R S, Electron Microscopy Sciences, Hatfield, PA) and studied in a direct mode using a JEOL 6060 scanning electron microscope (Oxford Instruments, UK).

### Cultured cells.

Human endothelial (EA.hy926, ATCC) and kidney epithelial (HEK293, ATCC) cells were cultured according to manufacturer instructions. Genetically engineered HEK 293 cells with a triple knockout of ORAI1, ORAI2, and ORAI3 store-operated Ca^2+^ entry (SOCE) channels generated using CRISPR-Cas9 gene editing as previously described^27^.

### Live cell Ca^2+^ imaging.

Ca^2+^ events were recorded in cells before and after histone stimulation using the Ca^2+^ indicators. HEK cells were loaded with Fura-2 and imaged with a plate reader. Cells were manually identified and Ca^2+^ levels within each cellular region of interest were reported as the ratio of F_340_/F_380_. ECs (EA.hy926 or mesenteric artery vascular preparations) were loaded with Cal-520 imaged using spinning disk confocal microscopy. Ca^2+^ oscillations from movies were analyzed using automated custom software (Volumetry, Grant Hennig) to create 2D and 3D prevalence maps allowing quantification of the numbers of cells activated per field and the frequency, amplitude, and duration of Ca^2+^ events, as previously described^19^. Ionomycin was applied at the conclusion of each experiment as a positive control which maximally increased Ca^2+^ levels in all cells within the field of view^33^.

### Live cell membrane imaging.

EA.hy926 cells were plated on glass coverslips and grown to ~70% confluence. The coverslips were transferred to individual wells containing HEPES-PSS with no added Ca^2+^, 1.2 mM Ca^2+^, or 12 mM Ca^2+^ as indicated. Membrane dye FM1–43 (2 μM) and Gd^3+^ (10 μM) – where indicated – were added directly to the wells immediately prior to imaging. A baseline 3D Z-stack was then acquired before the careful addition of 1 mL extra HEPES-PSS (vehicle control), 50 μg/mL histones or 10 μM ionomycin. Continuous 2D recordings were acquired at 1 frame per second for 40 minutes. Finally, a second Z-stack was obtained. Individual cell cytoplasm ROIs were drawn manually in ImageJ, excluding outer membrane signal at all timepoints. Fluorescence intensity was quantified for each cell as ΔF/F0 using the following formula: (F_t_ - F_0min_) / F_0min_. For representative Z-stack images, .png files were loaded into ImageJ and converted to 8-bit. Contrast was enhanced by lowering the maximum to 150 for each image within ImageJ.

### Live cell viability imaging.

EA.hy926 cells were seeded at a density of 800,000 on 12 mm glass coverslips and allowed to grow for 1–2 days prior to imaging. Hoechst 33342 (1 μg/mL) and SYTOX Green (50 nM) were added to each well and incubated for 10 minutes at 37 °C in 5% CO_2_. Coverslips were washed three times with HEPES-PSS following staining. For baseline and post-stim timepoints, three fields were imaged per coverslip, each consisting of a z-stack of 50 slices covering approximately 15–20 μm in depth. Following baseline imaging, coverslips were transferred into stimulation wells containing either HEPES-PSS control, histones (100 μg/mL) in HEPES-PSS, or ionomycin (10 μM) in HEPES-PSS. Cells were incubated at 37 °C for 1 hour. At 50 minutes, SYTOX Green was added to the stimulation wells and returned to the incubator. At the 1-hour mark, cells were imaged immediately (within 2–4 minutes), acquiring three non-overlapping fields per coverslip. After imaging, coverslips were washed and transferred into a recovery well pre-filled with 2 mL of media and returned to the incubator for a 3-hour recovery period. After the 3-hour recovery in media, coverslips were removed, washed with HEPES-PSS, and placed in fresh HEPES-PSS containing SYTOX AADvanced for 10 minutes before final imaging. For the final timepoint, three fields per coverslip were imaged using a reduced stack depth of three slices (~15 nm).

### Recovery Imaging Analysis.

Raw images were acquired using Fusion software and post-processed in Imaris. Standard parameters were applied uniformly across all replicates to ensure consistency, including four iterations of deconvolution and baseline subtraction using a 0.218 μm spatial threshold, as specified in the software’s baseline correction settings. Nuclear regions were identified based on the Hoechst 33342 signal. Using the Imaris classification tool, each nucleus was then assigned to a viability category – double-positive (SYTOX Green^+^/SYTOX AADvanced^+^), SYTOX Green^+^ only, SYTOX AADvanced^+^ only, or negative – based on fluorescence intensity and channel overlap within the defined nuclear region. This approach ensured each cell was counted once and accurately categorized based on nuclear uptake alone.

### Flow Cytometry.

Cultured ECs were plated in 24-well tissue culture plates. Once cells were grown to confluency, the media was aspirated off and replaced with HEPES-PSS buffer solution containing histones as indicated. HEPES-PSS solutions contained no added Ca^2+^, 1.2 mM Ca^2+^, or 12 mM Ca^2+^ depending on the condition. For membrane permeability assays, FM1–43 (2 μM) was added concurrently with histone exposure and incubated with the cells for 40 minutes at 37 °C with 5%. The cells were then trypsinized, centrifuged, and washed with phosphate buffered solution (PBS). For experiments evaluating cell viability, cells were trypsinized and washed with PBS then resuspended in Annexin V binding buffer and stained with Annexin V (Biolegend #640920) and Propidium Iodide (Thermo Fisher #2301111). Cells were incubated at room temperature for 15 minutes prior to 1:5 dilution in binding buffer. Samples were run on a flow cytometer (MACSQuant Analyzer 10, Miltenyi Biotec). Overall sample fluorescence was quantified as the median fluorescent intensity (MFI); n of 3–4 for all conditions.

### Protein-Lipid Binding Assay.

Mega Lipid Strips (Echelon-Inc, Salt Lake City, UT) kits were used in binding assays according to the manufacturer’s instructions. Briefly, strips were blocked for one hour at room temperature with 3% bovine serum albumin (BSA: Sigma-Aldrich, St. Louis, MO) in PBS-T (phosphate buffered saline with 1% Tween-20). Strips were then incubated with Histone H3-GST (0.5 μg/mL; BPS Biosciences, San Diego, CA) for one hour at room temperature on an orbital shaker (170 RPM). Strips were then washed in PBS-T three times for 7 minutes each wash. Strips were then incubated in 75 μL of a GST secondary antibody (Echelon-Inc, Salt Lake City, UT) for one hour at room temperature. Strips were washed twice in PBS-T and twice in PBS for 5 minutes each. Strips were then incubated in 2 mL of TMB precipitating solution (Echelon-Inc, Salt Lake City, UT) for 2 minutes before imaging. Gd^3+^ (30 μM) was added during the blocking step prior to the addition of histones where indicated. Following background subtraction, dot blot intensity was then quantified as area under the curve (AUC) using ImageJ software.

### Electric Cell-substrate Impedance Sensing.

EA.hy926 cells were grown to confluence at a density of 200,000 cells on 8-well polyethylene terephthalate (PET) electrode arrays (8W10E+, Applied Biophysics, Inc., Troy, NY) for 48 hours. Prior to seeding the electrode arrays were treated with 10 mM L-cysteine electrode-stabilizing solution for 15 minutes and allowed to air dry to provide a robust cleaning of the gold plating. Electric Cell-substrate Impedance Sensing (Model Z Theta, Applied BioPhysics, Inc., Troy, NY) data was collected from the capacitance values at 4,000 kHz over the course of 1.5 hours and normalized to the first measurement point.

### Data analysis and statistics.

GraphPad Prism software (ver. 10.4.0; GraphPad Software, USA) was used for x–y graphing and analysis. Data were tested for normality using the Kolmogorov-Smirnov test. Parametric t-test or ANOVA, or appropriate non-parametric alternatives were applied for comparisons between groups, and differences were considered significant if P < 0.05.

#### Ca^2+^ Fluorescence (Fura-2) Intensity.

Max peak intensity was quantified for each cell for the period of stimulation. Since multiple measurements were taken from the same cell and compared across multiple stimuli, matched values for each cell were stacked. For each trace, a two-way ANOVA was conducted to compare the means across WT and ORAI TKO for each period of stimulation. Geisser-Greenhouse correction was used for non-sphericity and Šidák correction was used to correct for multiple comparisons.

#### Spatiotemporal Analysis of Ca^2+^ Fluorescence (Cal-520).

Data was quantified as the total area with Ca^2+^ signals, normalized as a percent of the ionomycin response. Thus, each independent replicate generated a percentage for baseline, Gd^3+^, and histones. Two individual Friedman Tests for nonparametric, matched data were conducted for the cultured and *en face* preps respectively.

#### Lipid Binding.

The area under the curve (AUC) was quantified in ImageJ for each lipid dot. A two-way ANOVA was used to compare histone-lipid binding for each lipid across different ionic conditions (0 mM Ca^2+^, 12 mM Ca^2+^, 30 μM Gd^3+^). Data points were stacked by replicate (n=3). Geisser-Greenhouse correction was used for non-sphericity and Tukey correction was used to correct for multiple comparisons.

#### Membrane Imaging.

Cell fluorescence (FM1–43) at 40 minutes was quantified as ΔF_40min_/F0. All individual cell values were combined across replicates (n=3) and a Kruskal-Wallis test for non-parametric data was performed. A threshold of greater than 1.0 ΔF_40min_/F0, representing 2-fold increase in fluorescence relative to baseline, was used to calculate the percent of cells filled with dye at 40 minutes.

#### Flow Cytometry.

MFI was quantified using the median fluorescent value for each histone replicate (n=3 or 4) and compared across ionic conditions (0 mM, 1.2 mM, 12 mM Ca^2+^) using a Kruskal-Wallis test.

#### Electric Cell-substrate Impedance Sensing (ECIS).

Raw traces were normalized to the starting impendence at the time of histone treatment (time 0) so all traces aligned at 1.0. Endpoint resistance was taken at the very end of the experiment across all groups and compared using a t-Test. Total resistance area under the curve (AUC) for each run was automatically calculated using Graphpad Prism and compared using a t-test.

## Supplementary Material

Supplement 1 **Supplemental Figure 1. Low-dose Gd**^**3+**^ **pre-treatment only partially blocks histone-induced Ca**^**2+**^ **oscillations in HEK-293 cells, independent of ORAI channels.** Wildtype (WT, left) and ORAI triple knockout (TKO, right) HEK-293 cells were stained with Ca^2+^ indicator (Fura-2) and stimulated with histones (50 μg/mL, n=1). Ionomycin (10 μM) was added at the end of each experiment as a positive control. (A) Representative traces of fluorescence over time in response to pretreatment with Gd^3+^ (5 μM) followed by histones (50 μg/mL) for WT (left) and ORAI-TKO (middle). Quantification of maximum peak intensity for each cell (right). (B) Representative traces of fluorescence over time in response to post-treatment with Gd^3+^ (5 μM) followed by histones (50 μg/mL) for WT (left) and ORAI-TKO (middle). Quantification of maximum peak intensity for each cell (right). (C) Representative traces of fluorescence over time in response to histones (50 μg/mL) in 0 mM Ca^2+^ followed by 2 mM Ca^2+^ for WT (left) and ORAI-TKO (middle). Quantification of maximum peak intensity for each cell (right). 2-way ANOVA for significance (n = 1 for each experiment).

Supplement 2 **Supplemental Figure 2. Moderate dose histones induce a subtle, pre-apoptotic phenotype in ECs, which is enhanced by low Ca**^**2+**^ **enhances and blocked by high Ca**^**2+**^ **blocks.** EA.hy926 cells were treated with histones (50 μg/mL) in HEPES-PSS with 0, 1.2, or 12 mM Ca^2+^ for 1 hour, followed by Annexin V (PS) and PI staining. (A) Representative flow cytometry histograms showing fluorescence of Annexin V (top) and PI (bottom) for cells incubated in HEPES-PSS alone (blue) or with the addition of histones (pink). (B, C) Median fluorescence intensity (MFI) for Annexin V and PI shown (n = 4). (D) Representative dot plots of Annexin V and PI fluorescence. (E, F) Quantification of percent PS^+^/PI^−^ cells and PS^+^/PI^+^ cells using the gating strategy shown in D.

Supplement 3 **Supplemental Figure 3. Histones disrupt endothelial cell monolayer barrier integrity and cause a significant decrease in electrical resistance in a low Ca2+ environment.** (A) Raw traces from Electric Cell-substrate Impedance Sensing (ECIS) measurements. Histone (100 μg/mL) treatment caused a rapid decrease in endothelial cell monolayer resistance in low Ca^2+^ HEPES buffer versus monolayers in normal (1.2 mM) Ca^2+^ HEPES. Histone (100 μg/mL) treatment caused a significant difference in (B) normalized endpoint resistance between the normal and low Ca^2+^ HEPES buffer conditions (1.7 ± 0.1 Ω n=8 vs 0.5 ± 0 Ω n=7) as well as a significant difference in (C) total resistance between the same groups (AUC; 1558 ± 81 Ω n=8 vs 462 ± 9 Ω n=7).

Supplement 4 **Supplemental Video 1.** Histones induce Ca^**2+**^ influx in en face murine ECs. 3D representation of cumulative Ca^2+^ oscillations over the 5 minute period after exposure to histones (50 μg/mL). Spatio-temporal representation overlaid on 2D image of the same field of view with ionomycin-induced maximal Ca^2+^ response.

Supplement 5 **Supplemental Video 2.** Pretreatment with Gd^3+^ blocks histone-induced Ca^2+^ influx in en face murine ECs. 3D representation of cumulative Ca^2+^ oscillations after exposure to histones (50 μg/mL) in the presence of Gd^3+^ (10 μM). Spatio-temporal representation overlaid on 2D image of the same field of view with ionomycin-induced maximal Ca^2+^ response.

Supplement 6 **Supplemental Video 3.** Video recording (25x) of ionomycin-induced endothelial cell membrane changes visualized using the membrane dye FM1–43.

Supplement 7 **Supplemental Video 4.** Video recording (25x) of histone-induced endothelial cell membrane deformation visualized using the membrane dye FM1–43.

Supplement 8 **Supplemental Video 5.** Full field of view with inset video of endothelial cell filopodia extrusion and movement after application of ionomycin (30 minute recording: speed 96x).

Supplement 9 **Supplemental Video 6.** Full field of view with inset video of endothelial cell membrane blebbing after application of ionomycin (30 minute recording, speed 96x).

Supplement 10 **Supplemental Video 7.** Full field of view with inset video of endothelial cell membrane ruffles after application of histones (20 minute recording, speed 96x).

Supplement 11 **Supplemental Video 8.** Full field of view with inset video of endothelial cell rounding after application of histones (20 minute recording, speed 96x).

Supplement 12 **Supplemental Video 9.** Full field of view with inset video of endothelial cell membrane blebbing after application of histones (14 minute recording, speed 96x).

Supplement 13 **Supplemental Video 10.** Video of histones-induced endothelial cell membrane ruffling (30 minute recording, speed 96x).

Supplement 14 **Supplemental Video 11.** Video of histone-induced endothelial cell membrane blebbing (30 minute recording, speed 96x).

Supplement 15 **Supplemental Video 12.** Video of ionomycin-induced release and reuptake of extracellular vesicles (30 minute recording, speed 96x).

## Figures and Tables

**Figure 6.**
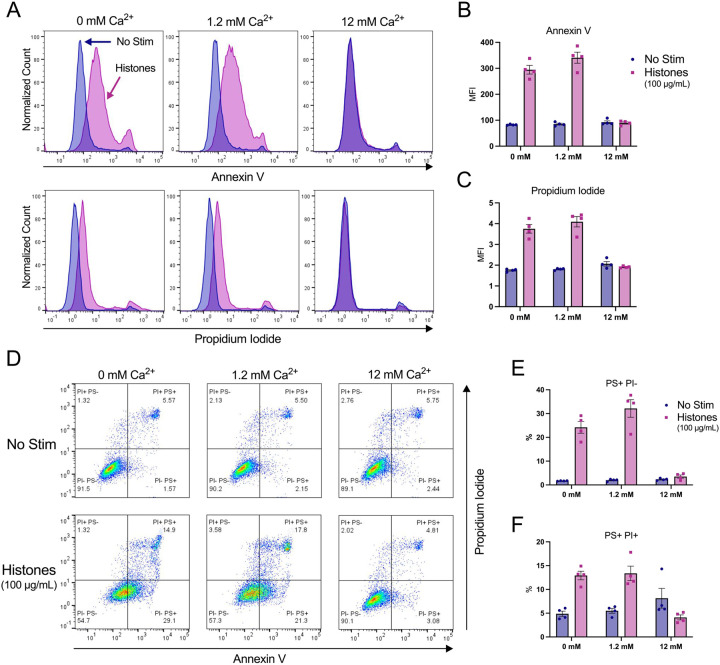
High dose histone exposure induces a pre-apoptotic phenotype in ECs, which is enhanced by low Ca^2+^ and blocked by high Ca^2+^. EA.hy926 cells were treated with histones (100 μg/mL) in HEPES-PSS with 0, 1.2, or 12 mM Ca^2+^ for 1 hour, followed by Annexin V (PS) and Propidium Iodide (PI) staining. (A) Representative flow cytometry histograms showing fluorescence of Annexin V (top) and PI (bottom) for cells incubated in HEPES-PSS alone (blue) or with the addition of histones (pink). (B, C) Median fluorescence intensity (MFI) for Annexin V and PI shown (n = 4). (D) Representative dot plots of Annexin V and PI fluorescence. (E, F) Quantification of percent PS^+^/PI^−^ cells and PS^+^/PI^+^ cells using the gating strategy shown in D.

## Significance

In acute critical illness, the rapid collapse of vascular endothelial functions drives aberrant blood clotting and organ failure through mechanisms that are not understood. Emerging evidence that early administration of donor plasma improves survival of trauma patients has transformed the massive transfusion protocols used in surgical settings, but the sodium citrate included in transfused blood products to prevent coagulation often produces significant and severe hypocalcemia. Here, we demonstrate that cytotoxic trauma factors that are elevated in the blood during resuscitation interact electrostatically with endothelial cell phospholipids, and that low Ca^2+^ exacerbates toxicity by increasing this interaction. Using high speed video imaging, we demonstrate fast endothelial cell membrane movements in response to injury, including protrusion and ruffling of lamellipodia, release and reuptake of extracellular vesicles, and blebbing. These findings provide important insights into the nature of shock-induced endotheliopathy and highlight the potential cardiovascular risk associated with chelation-induced hypocalcemia during resuscitation.
