# Supplementary figures and images for: Endothelial Trauma Depends on Surface Charge and Extracellular Calcium Levels
Source: bioRxiv. 2025 Sep 9:2025.07.13.664578. Originally published 2025 Jul 18. Preprint. [Version 4] doi: 10.1101/2025.07.13.664578 (PMC12338662; doi:10.1101/2025.07.13.664578)

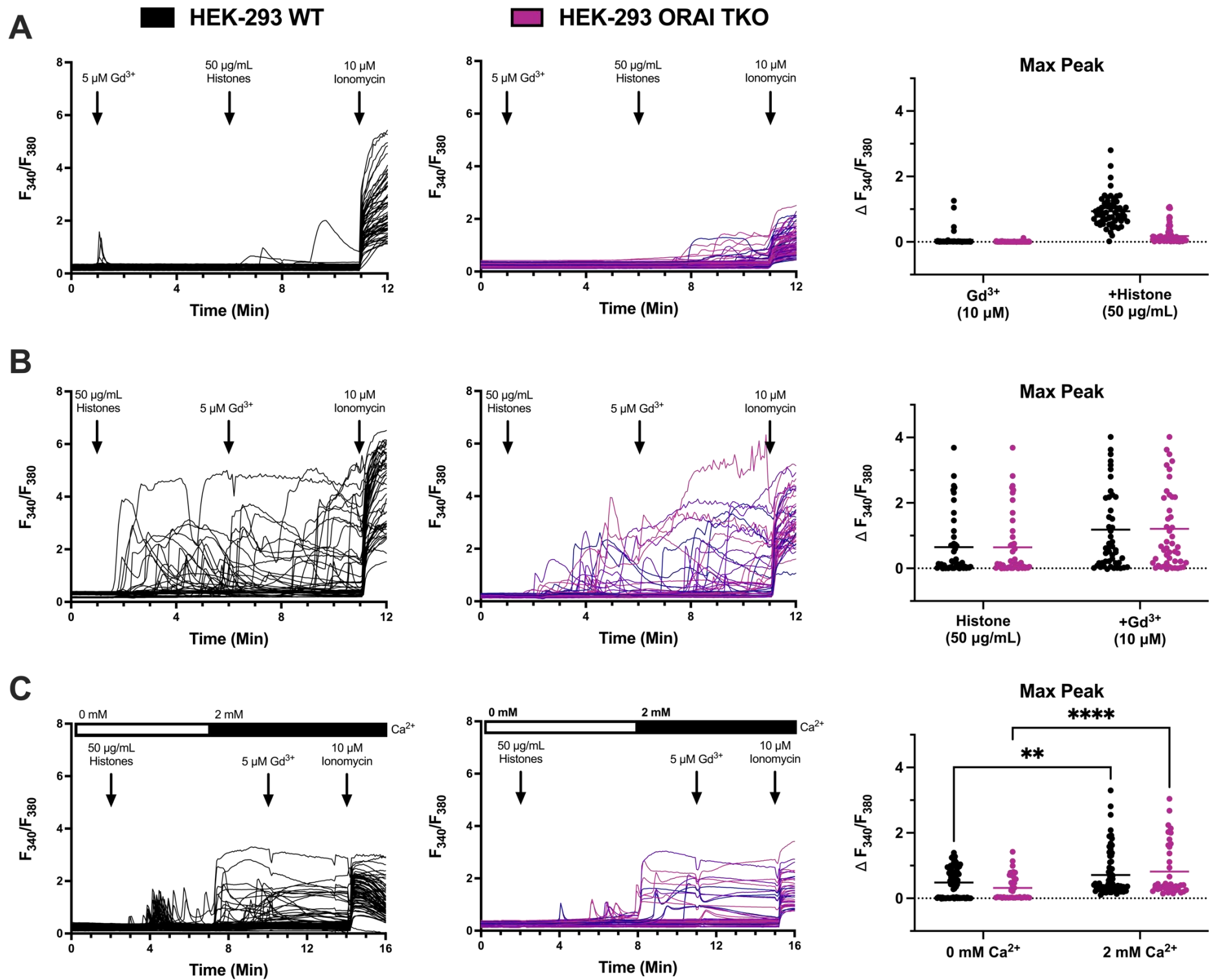

Supplement: Supplement 1 — Wildtype (WT, left) and ORAI triple knockout (TKO, right) HEK-293 cells were stained with Ca2+ indicator (Fura-2) and stimulated with histones (50 μg/mL, n=1). Ionomycin (10 μM) was added at the end of each experiment as a positive control. (A) Representative traces of fluorescence over time in response to pretreatment with Gd3+ (5 μM) followed by histones (50 μg/mL) for WT (left) and ORAI-TKO (middle). Quantification of maximum peak intensity for each cell (right). (B) Representative traces of fluorescence over time in response to post-treatment with Gd3+ (5 μM) followed by histones (50 μg/mL) for WT (left) and ORAI-TKO (middle). Quantification of maximum peak intensity for each cell (right). (C) Representative traces of fluorescence over time in response to histones (50 μg/mL) in 0 mM Ca2+ followed by 2 mM Ca2+ for WT (left) and ORAI-TKO (middle). Quantification of maximum peak intensity for each cell (right). 2-way ANOVA for significance (n = 1 for each experiment). [file media-1.pdf]

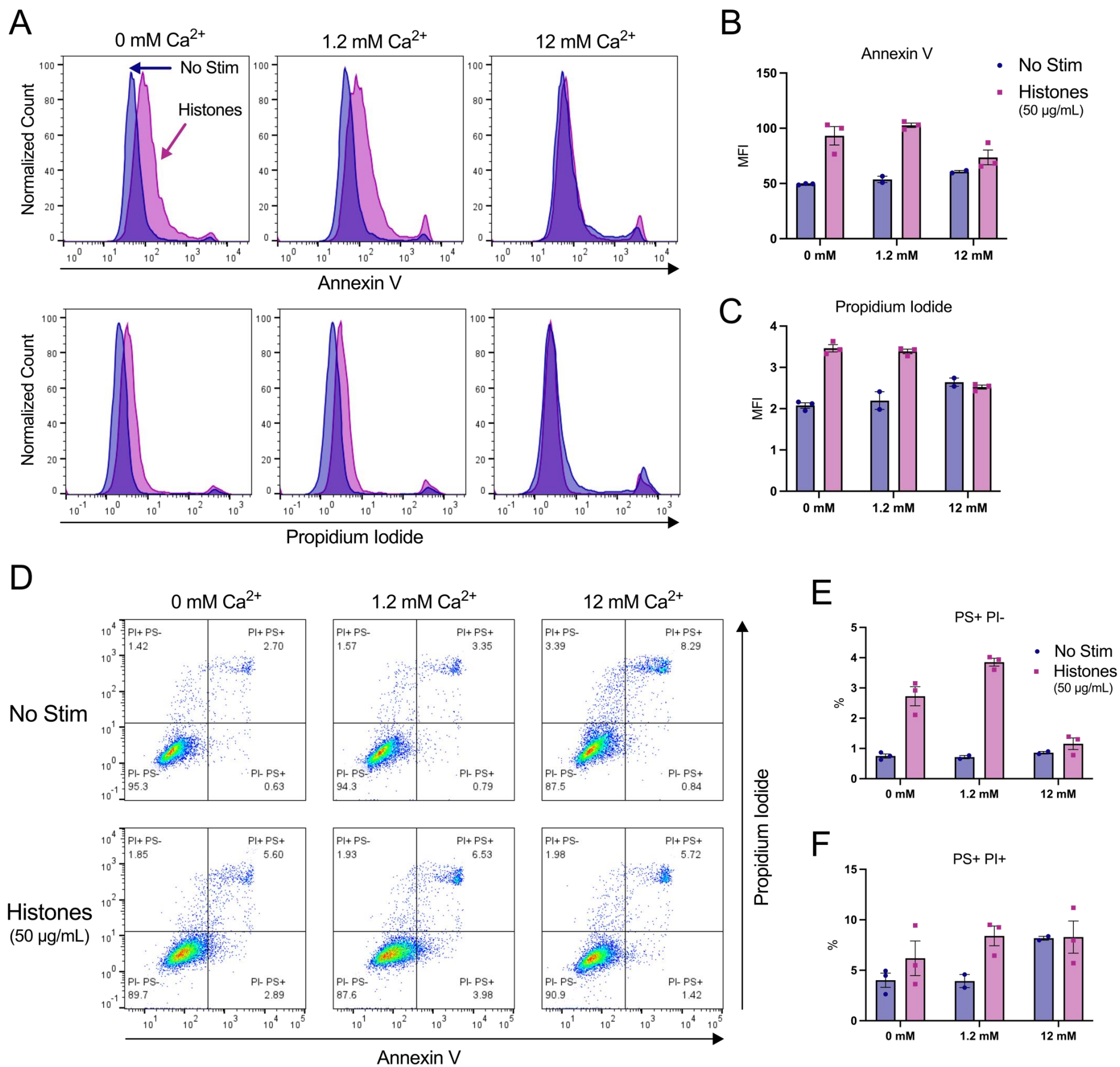

Supplement: Supplement 2 — EA.hy926 cells were treated with histones (50 μg/mL) in HEPES-PSS with 0, 1.2, or 12 mM Ca²+ for 1 hour, followed by Annexin V (PS) and PI staining. (A) Representative flow cytometry histograms showing fluorescence of Annexin V (top) and PI (bottom) for cells incubated in HEPES-PSS alone (blue) or with the addition of histones (pink). (B, C) Median fluorescence intensity (MFI) for Annexin V and PI shown (n = 4). (D) Representative dot plots of Annexin V and PI fluorescence. (E, F) Quantification of percent PS+/PI− cells and PS+/PI+ cells using the gating strategy shown in D. [file media-2.pdf]

A

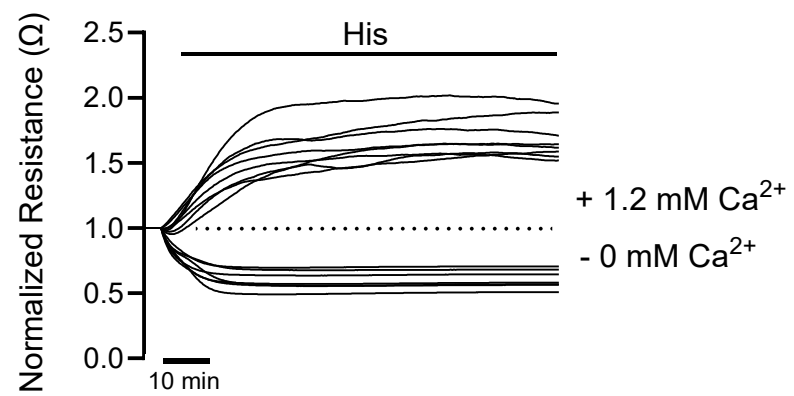

B

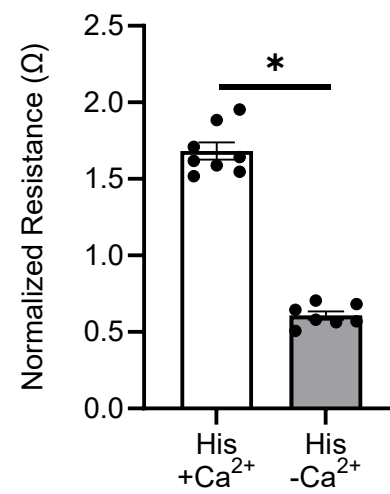

C

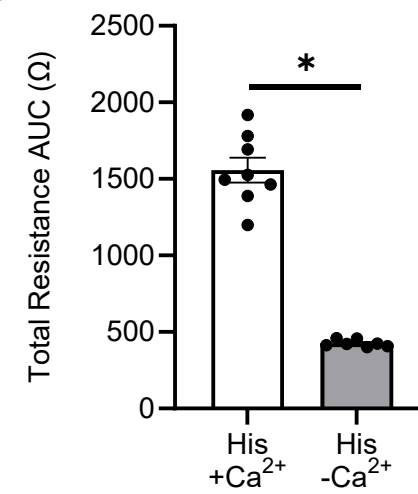

Supplement: Supplement 3 — (A) Raw traces from Electric Cell-substrate Impedance Sensing (ECIS) measurements. Histone (100 μg/mL) treatment caused a rapid decrease in endothelial cell monolayer resistance in low Ca2+ HEPES buffer versus monolayers in normal (1.2 mM) Ca2+ HEPES. Histone (100 μg/mL) treatment caused a significant difference in (B) normalized endpoint resistance between the normal and low Ca2+ HEPES buffer conditions (1.7 ± 0.1 Ω n=8 vs 0.5 ± 0 Ω n=7) as well as a significant difference in (C) total resistance between the same groups (AUC; 1558 ± 81 Ω n=8 vs 462 ± 9 Ω n=7). [file media-3.pdf]
